# Supplementary material for: Chemodiversity and Anti-Leukemia Effect of Metabolites from Penicillium setosum CMLD 18
Source: Metabolites. 2022 Dec 23;13(1):23. doi: 10.3390/metabo13010023 (PMC9864219; doi:10.3390/metabo13010023)
Supplement: Supplementary file 1 [file metabolites-13-00023-s001.zip › metabolites-2062873-supplementary.pdf]

Supplementary material

CHEMODIVERSITY AND ANTI-LEUKEMIA EFFECT OF METABOLITES FROM *Penicillium setosum*

Ana Calheiros de Carvalho [1], Cauê Santos Lima [2], Heron Fernandes Vieira Torquato [3], André Tarsis Domiciano [2], Sebastião da Cruz Silva [4], Lucas Magalhães de Abreu [5], Miriam Uemi [6], Edgar Julian Paredes-Gamero [7], Paulo Cezar Vieira [8], Thiago André Moura Veiga [6,*,†] and Lívia Soman de Medeiros [6,*,†]

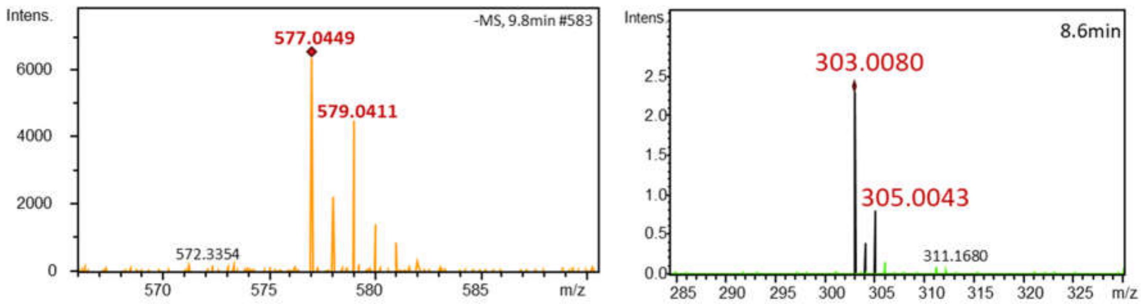

Figure S1: Mass spectrum (ESI-) of some peaks found in the base peak chromatogram obtained from the methanolic extracts of *Penicillium setosum* CMLD 18 (9.8 min.; 8.6 min.).

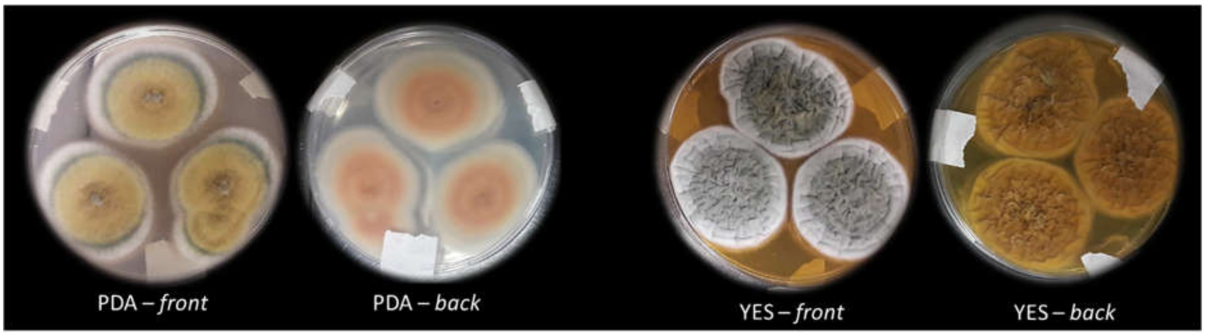

Figure S2. Macroscopic pictures of *Penicillium setosum* CMLD 18 in PDA and YES growth medium respectively.

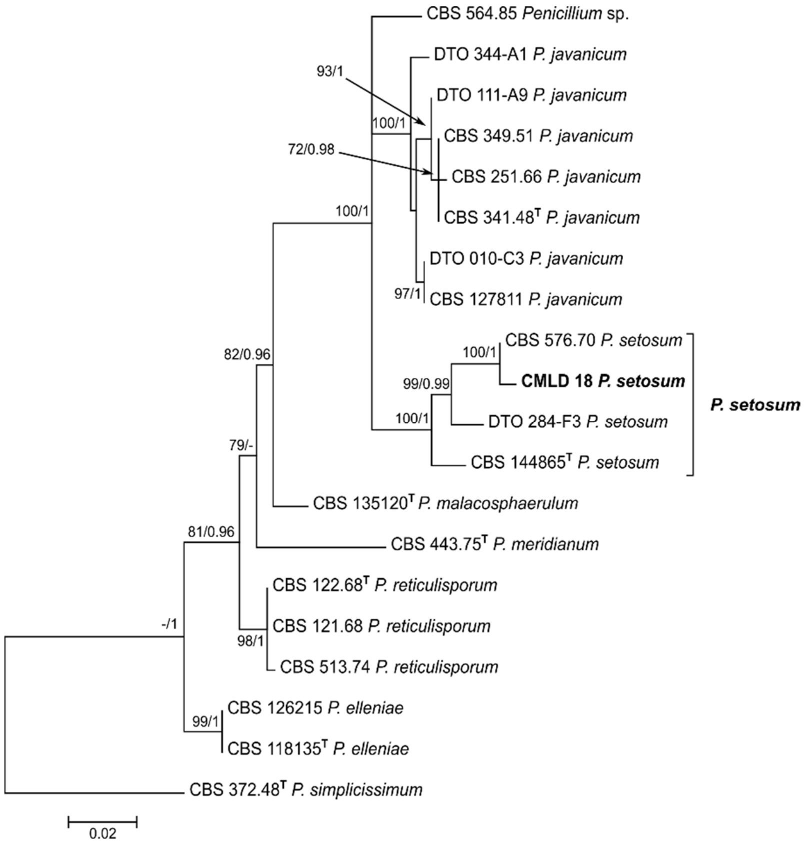

Figure S3: Bayesian Inference and Maximum Likelihood phylogenetic analyses of partial beta tubulin gene sequences placed the strain CMLD 18 inside a well-supported clade composed of the ex-type and other reference strains of *Penicillium setosum*. Morphological characterization of CMLD 18 using standard media and conditions used in *Penicillium* taxonomy (Visagie et al. 2014) supported the identification (data not shown).

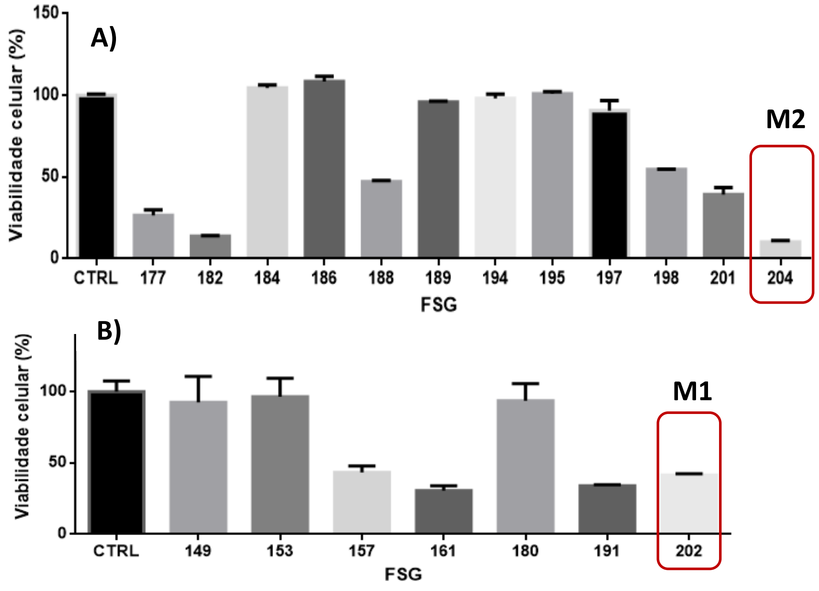

Figure S4: Cell viability of the methanolic fraction obtained from *P. setosum* cultivated in rice (A, M2) and hominy (B, M1) (highlighted in red) against the cell line KG-1 and Kasumi-1, respectively.

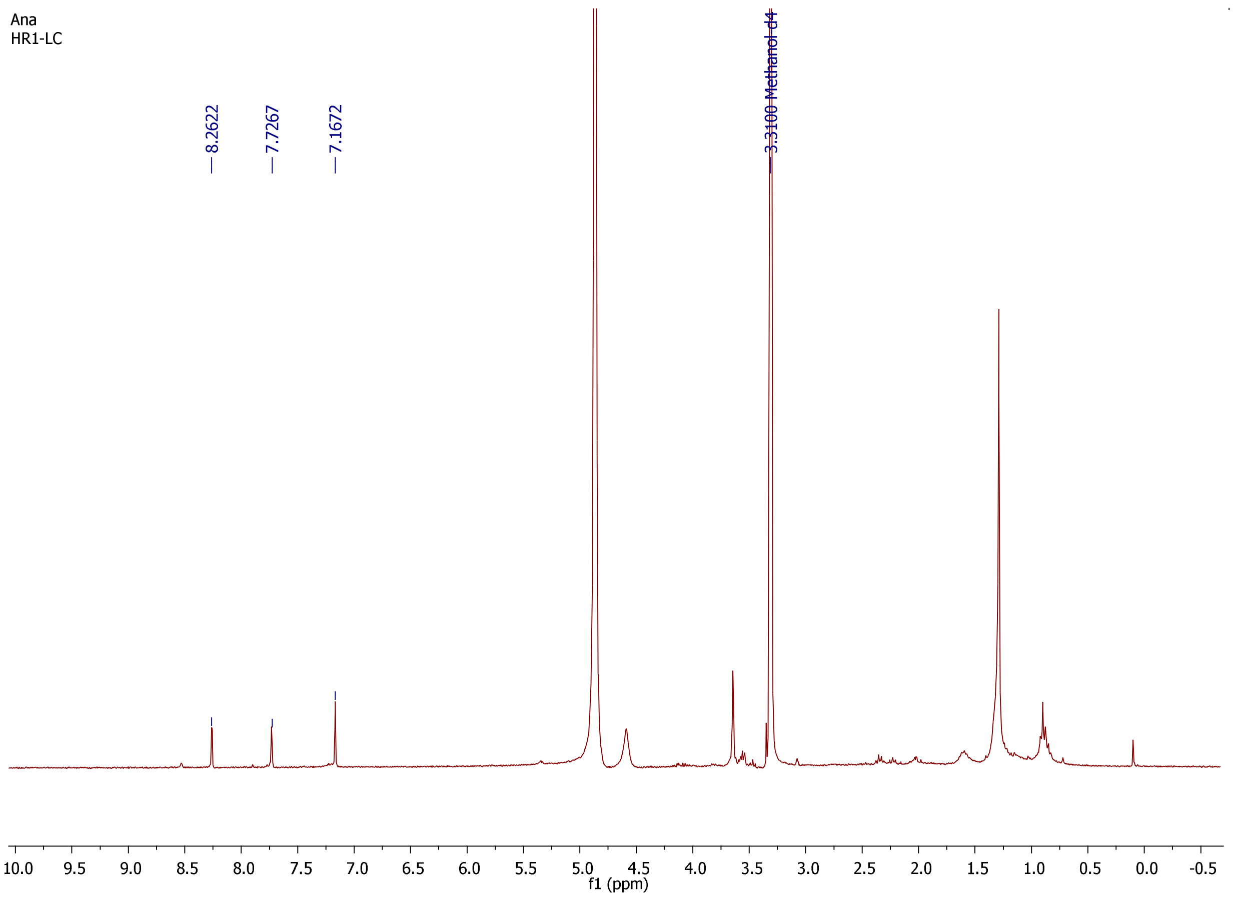

Figure S5: ¹H NMR spectrum of 2-chloroemodic acid (2) (300 MHz, methanol-*d4*).

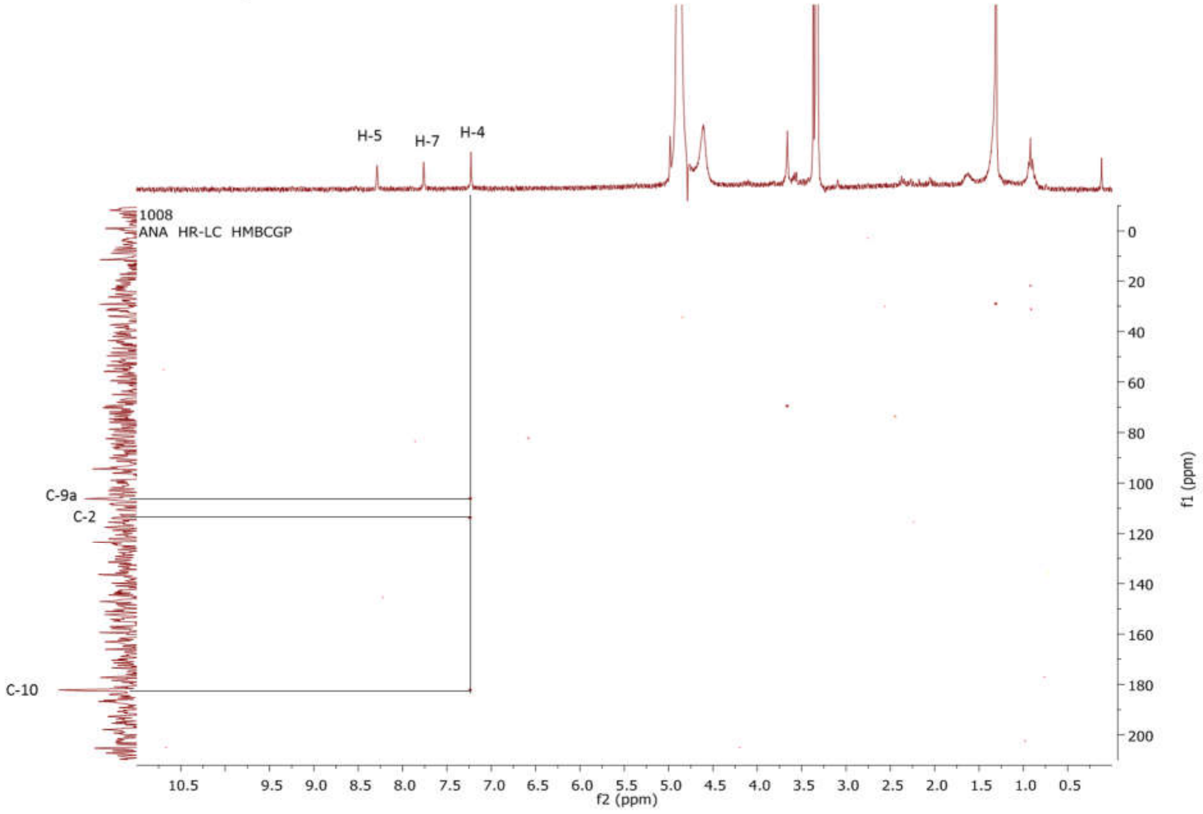

Figure S6: HMBC spectrum of 2-chloroemodic acid (2) (300 MHz, methanol-*d4*).

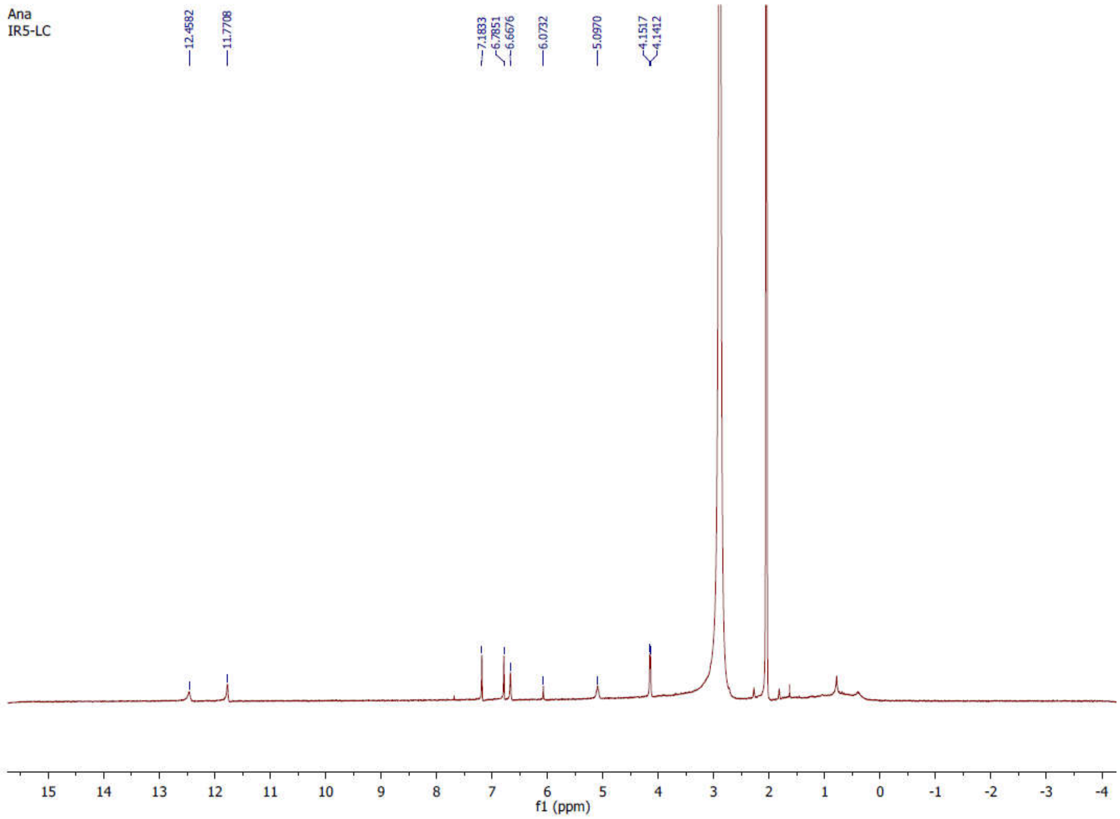

Figure S7: ^{1}H NMR spectrum of 2-chloro-1,3,8-trihydroxy-6-(hydroxymethyl)-anthraquinone (7) (300 MHz, acetone-d_6).

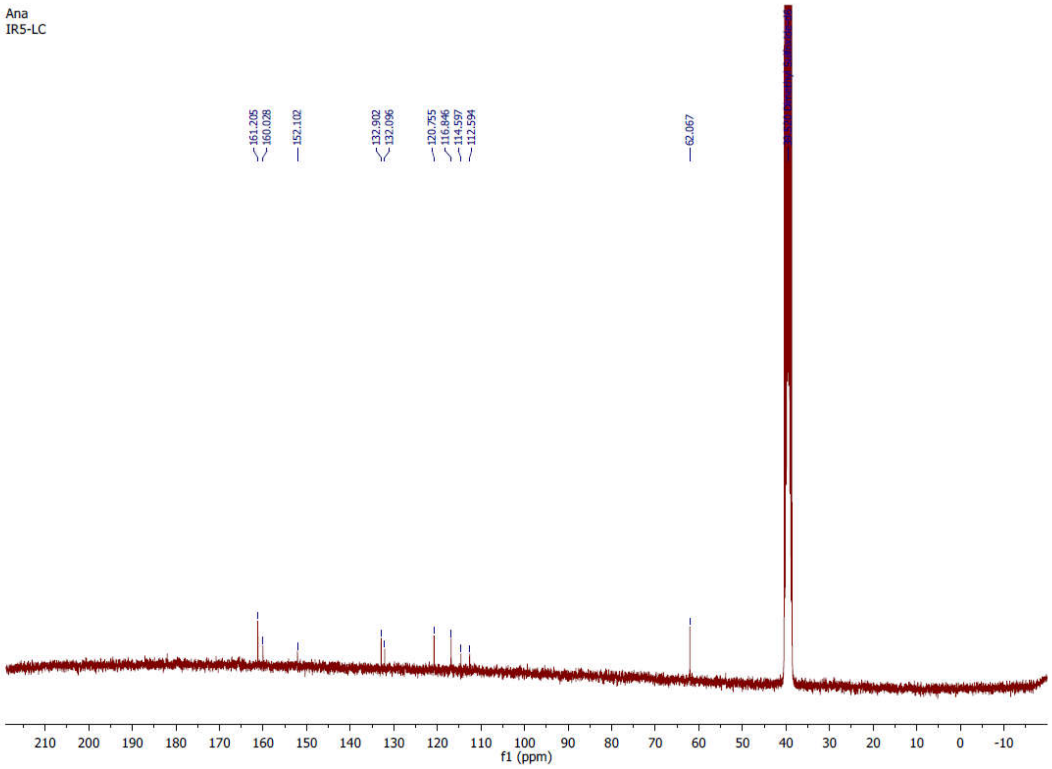

Figure S8: ^{13}C NMR spectrum of 2-chloro-1,3,8-trihydroxy-6-(hydroxymethyl)-anthraquinone (7) (300 MHz, DMSO-d_6).

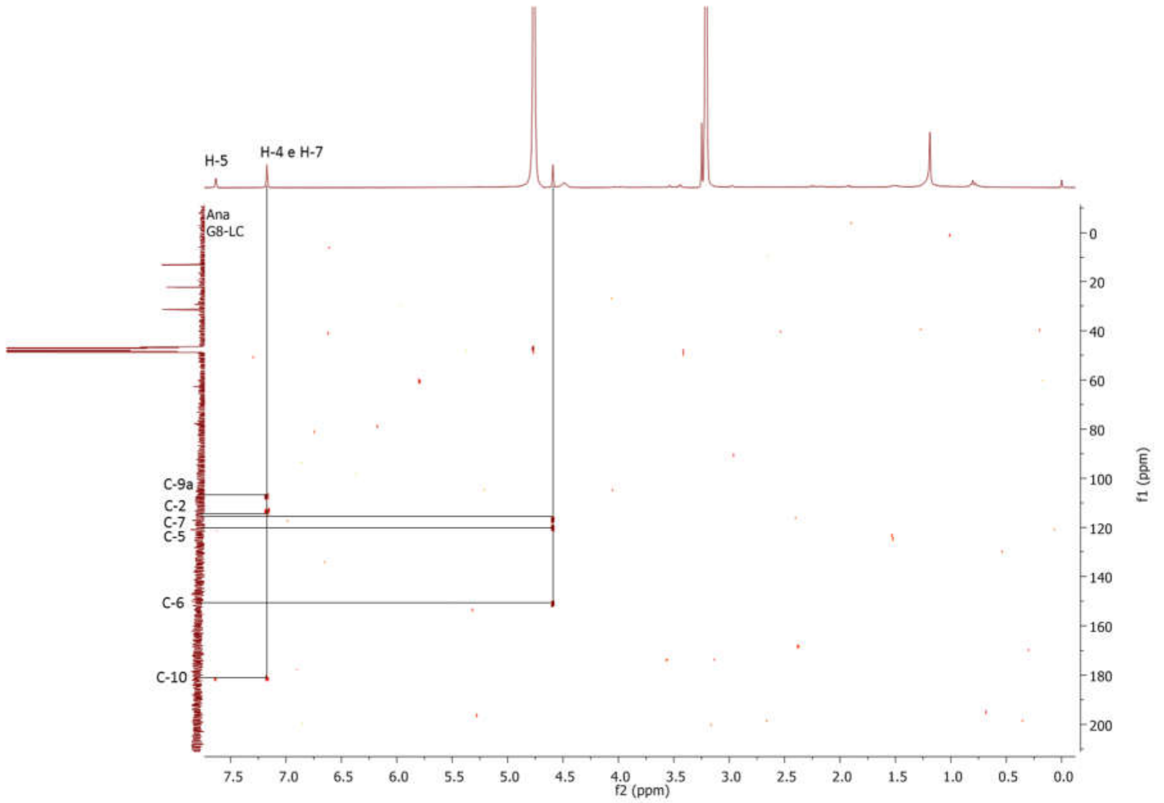

Figure S9: HMBC spectrum of 2-chloro-1,3,8-trihydroxy-6-(hydroxymethyl)-anthraquinone (7) (300 MHz, methanol-d4).

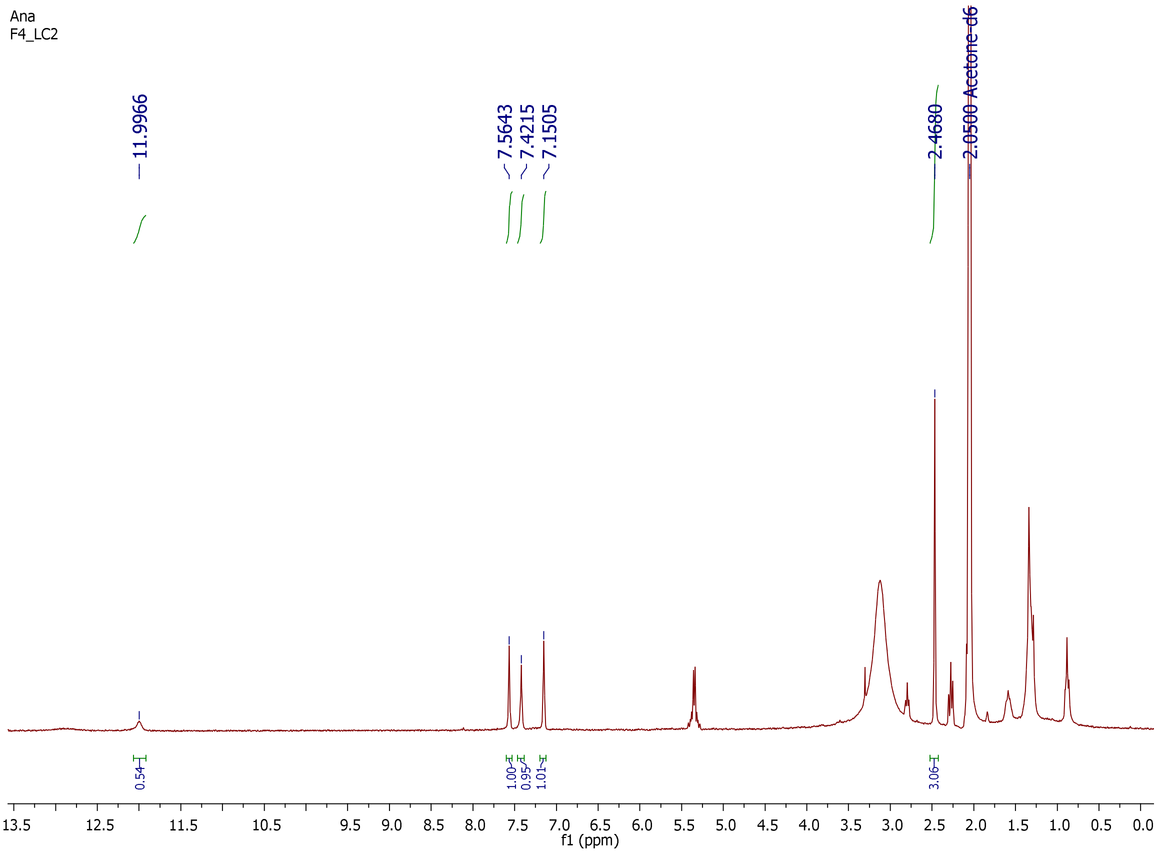

Figure S10: ^{1}H NMR spectrum of 7-chloroemodin (8) (300 MHz, methanol-*d4*)

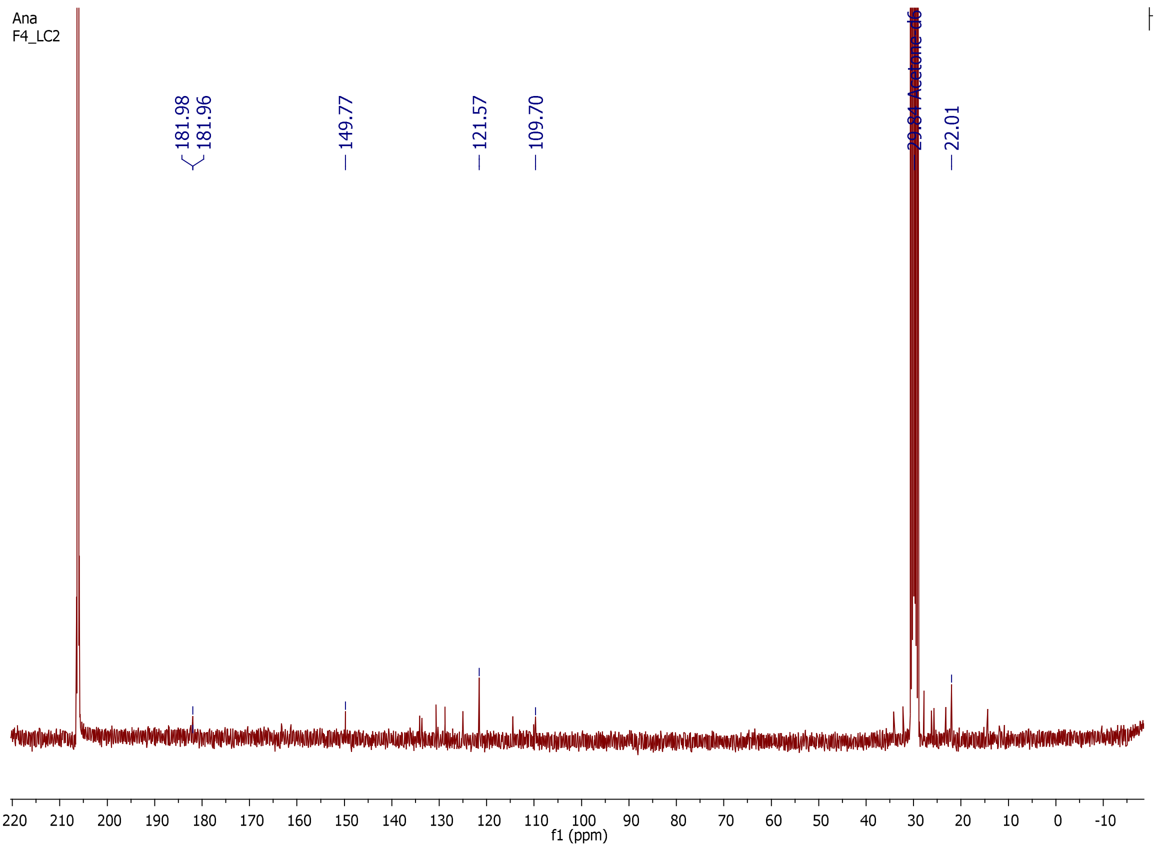

Figure S11: ^{13}C NMR spectrum of 7-chloroemodin (8) (300 MHz, methanol-*d4*)

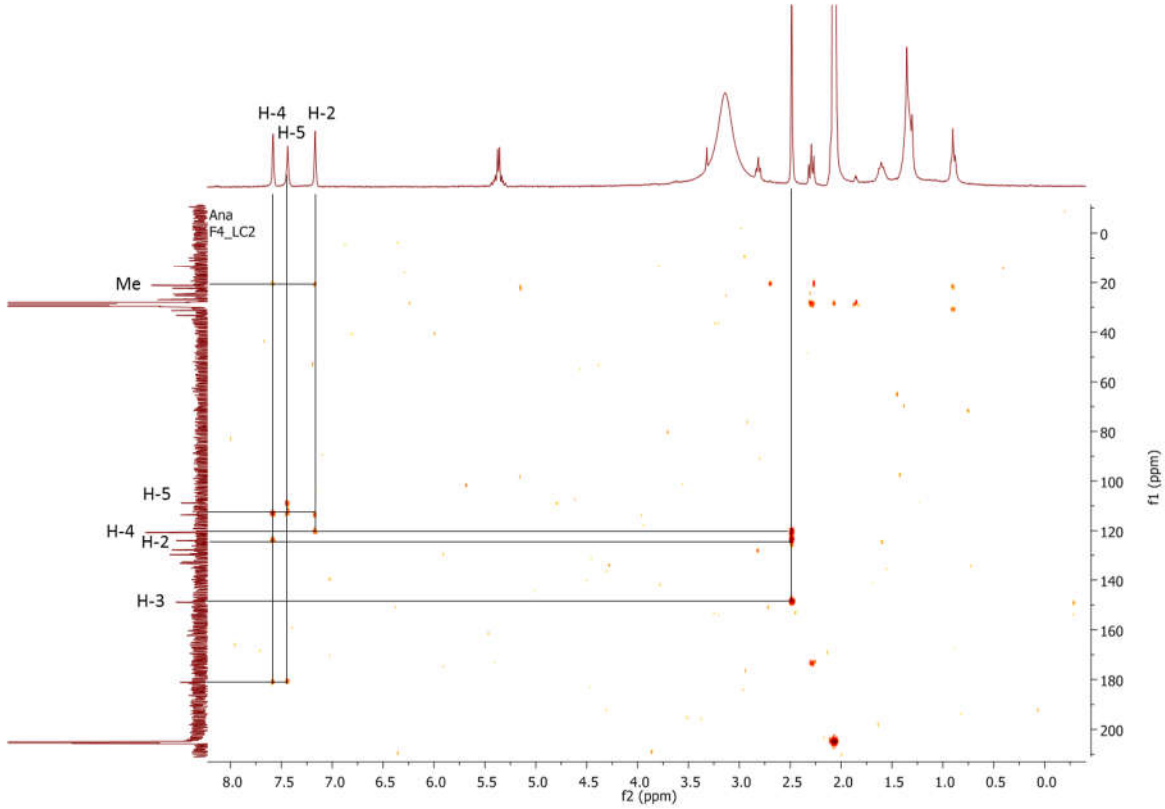

.

Figure S12: HMBC spectrum of 7-chloroemodin (8) (300 MHz, methanol-*d4*).

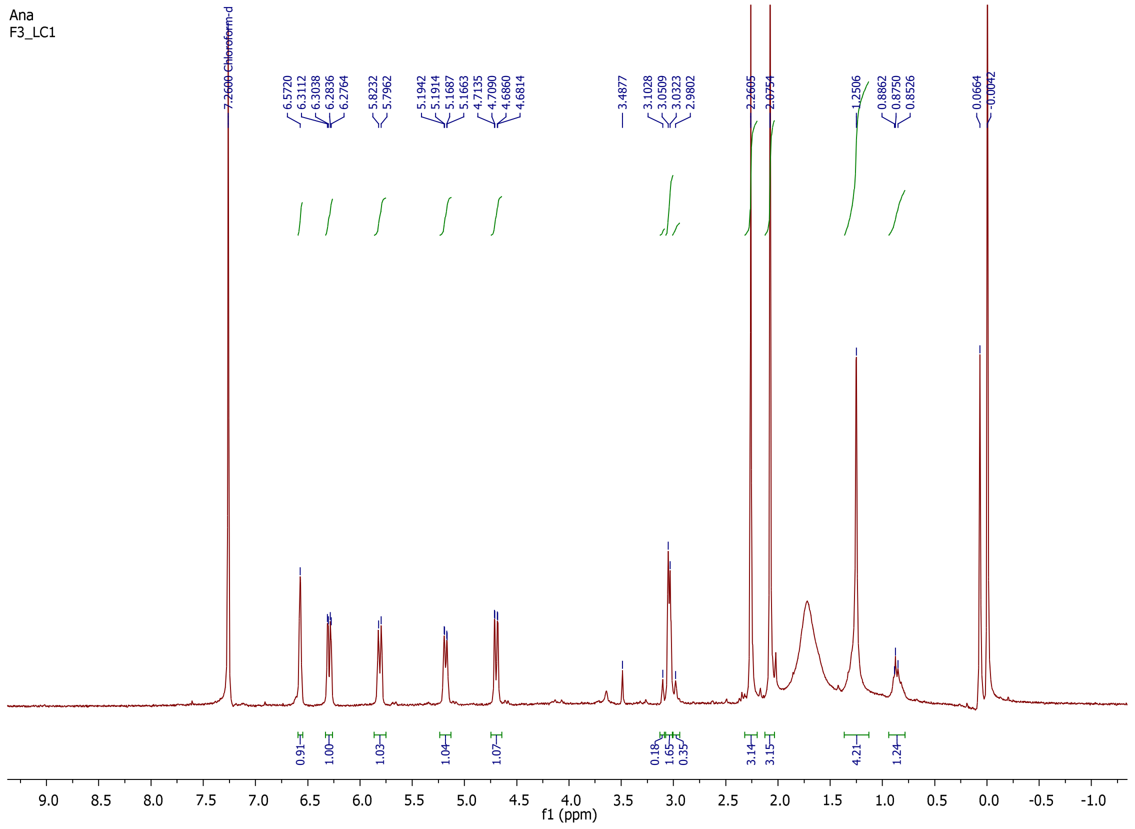

Figure S13: ^{1}H NMR spectrum of bisdethiobis(methylthio)acetylaranotine (9) (300 MHz, $CDCl_3$)

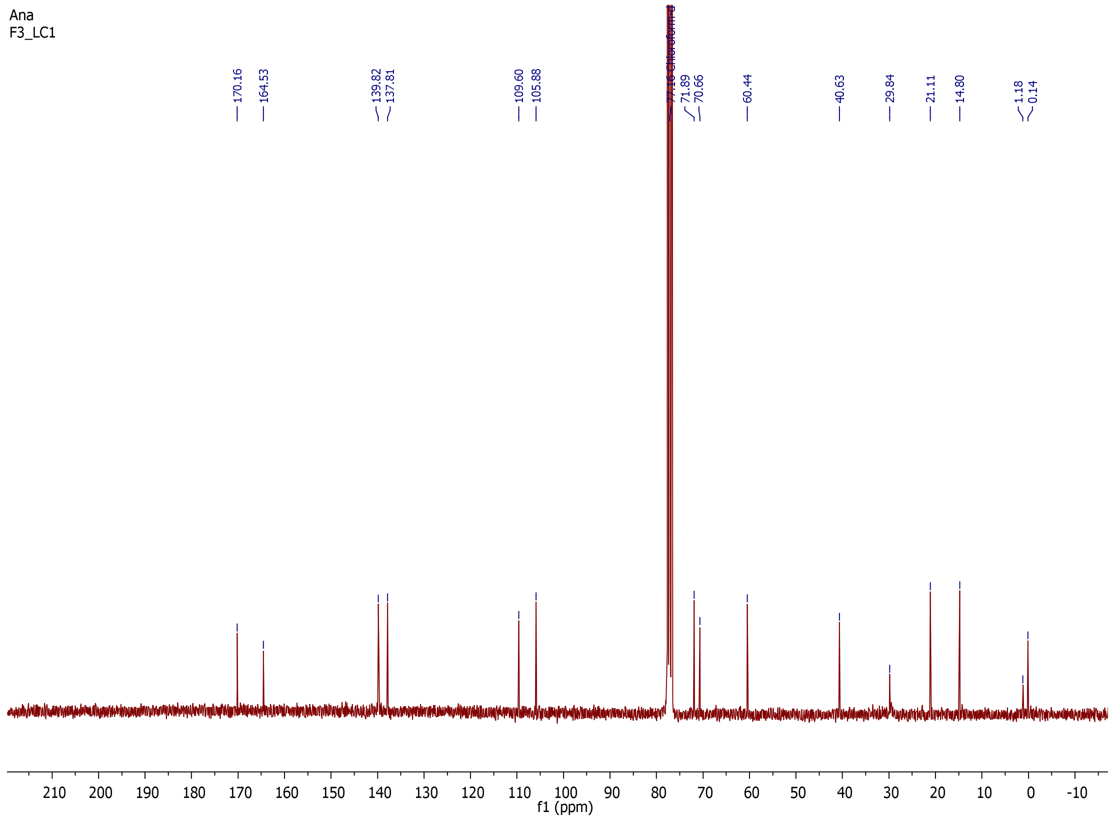

Figure S24: ^{13}C NMR spectrum of bisdethiobis(methylthio)acetylaranotine (9) (75 MHz, $CDCl_3$)

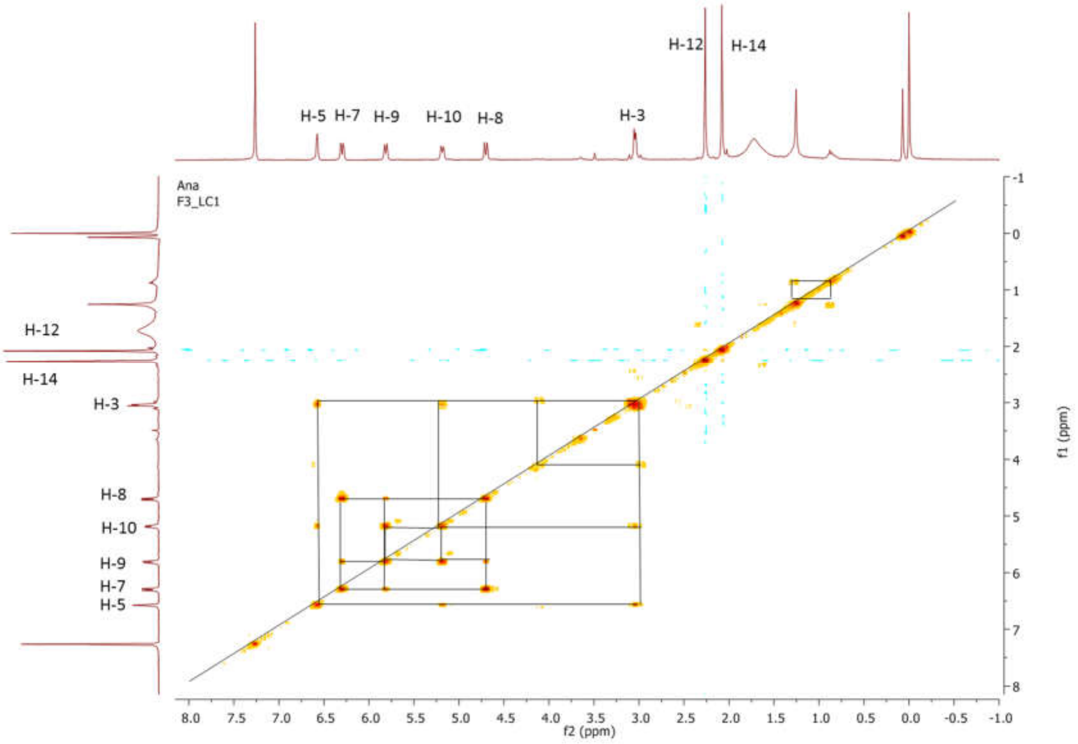

Figure S15: COSY spectrum of bisdethiobis(methylthio)acetylaranotine (9) (300 MHz, $CDCl_3$)

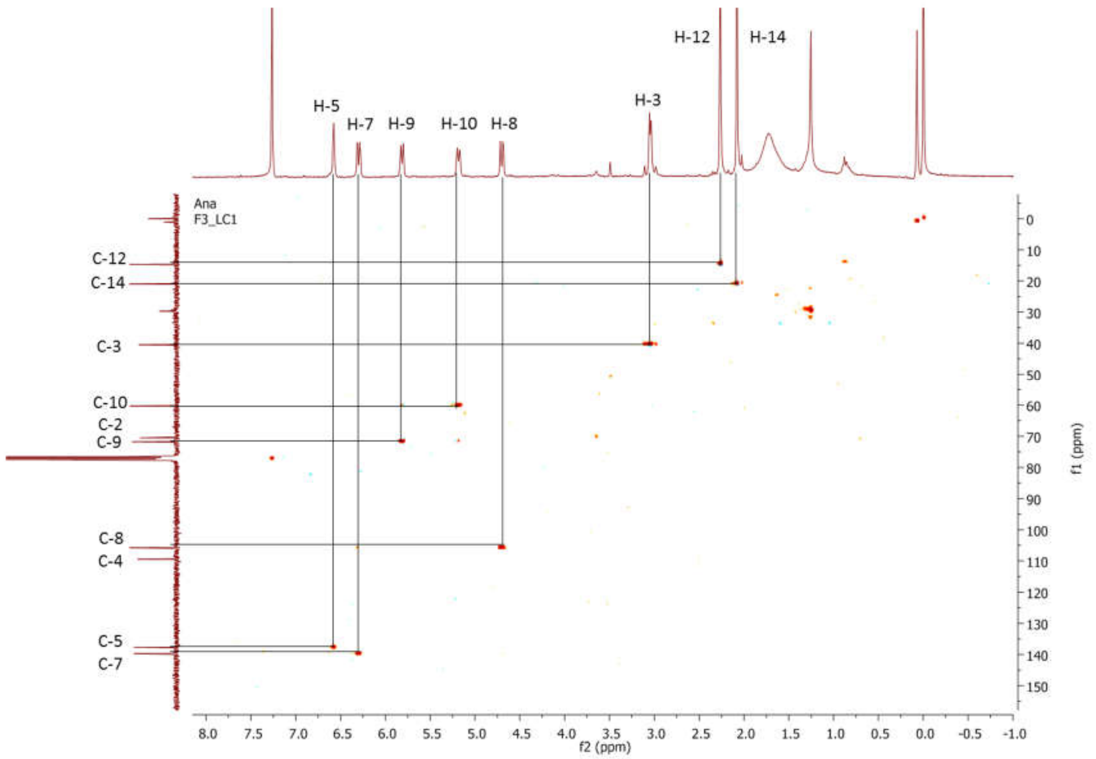

Figure S16: HSQC spectrum of bisdethiobis(methylthio)acetylaranotine (9) (300 MHz, $CDCl_3$)

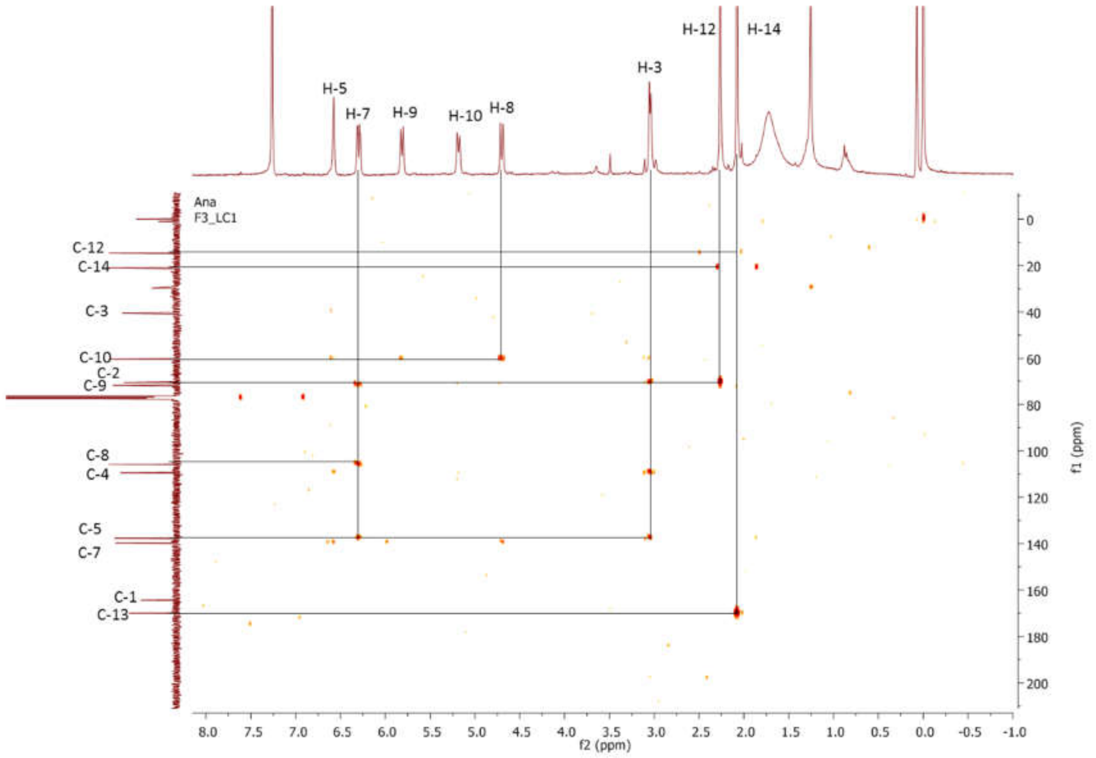

Figure S17: HMBC spectrum of bisdethiobis(methylthio)acetylaranotine (9) (300 MHz, $CDCl_3$)

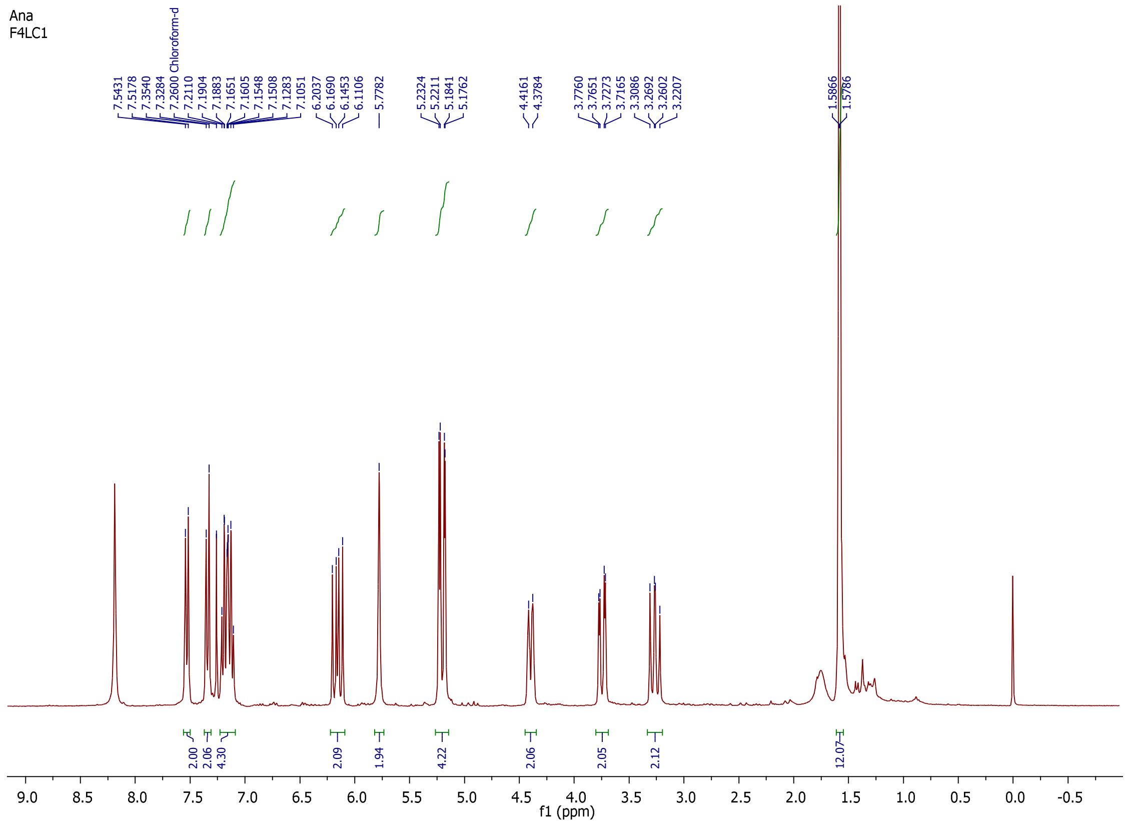

Figure S18: ^{1}H NMR spectrum of fellutanine C (10) (300 MHz, $CDCl_3$).

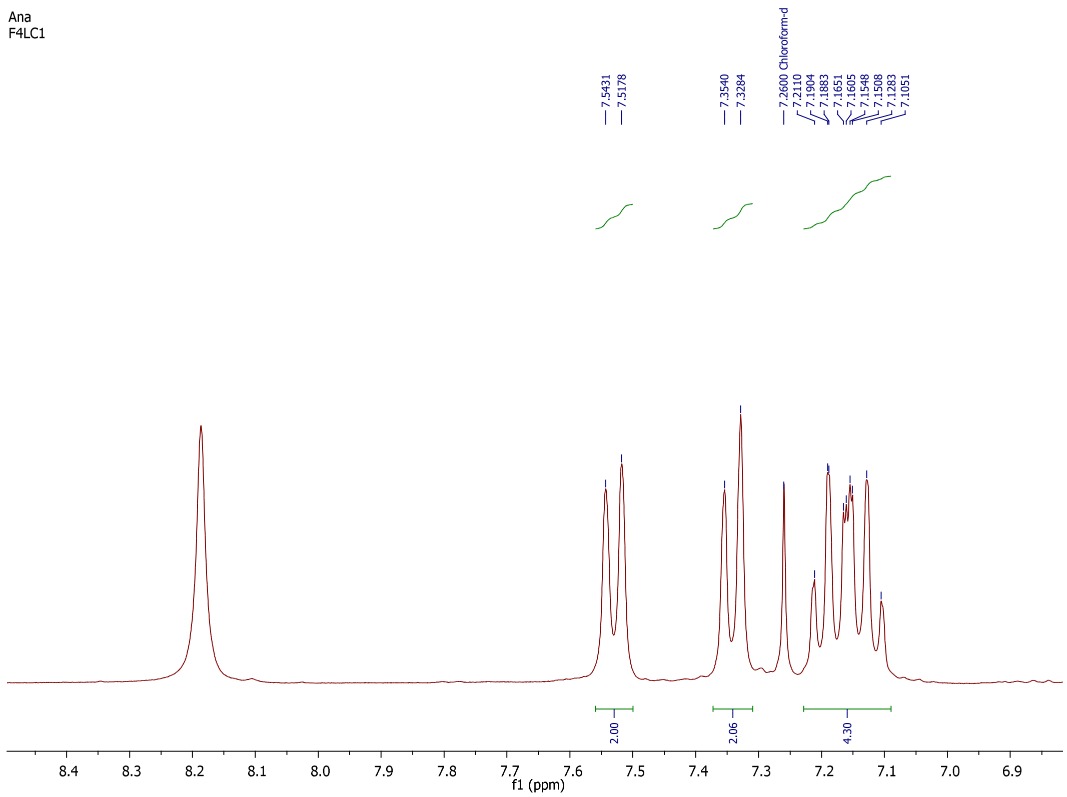

Figure S19: Magnified region from the 1H NMR spectrum of fellutanine C (10) (300 MHz, $CDCl_3$).

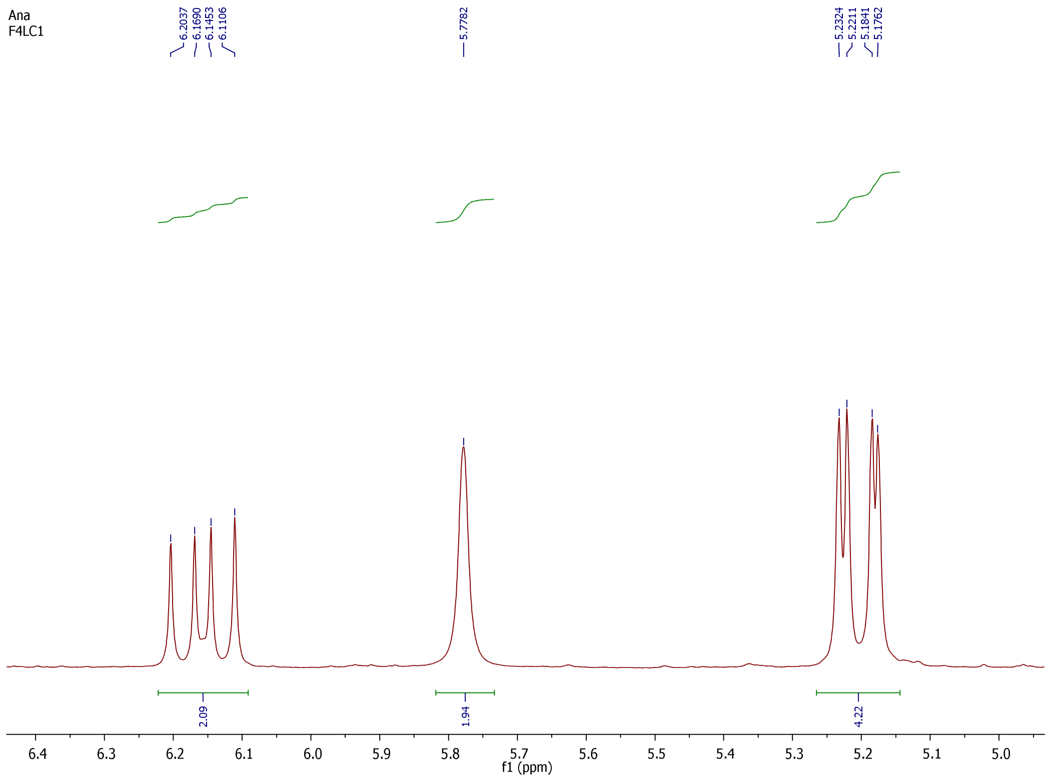

Figure S20: Magnified region from the ^{1}H NMR spectrum of fellutanine C (10) (300 MHz, $CDCl_3$).

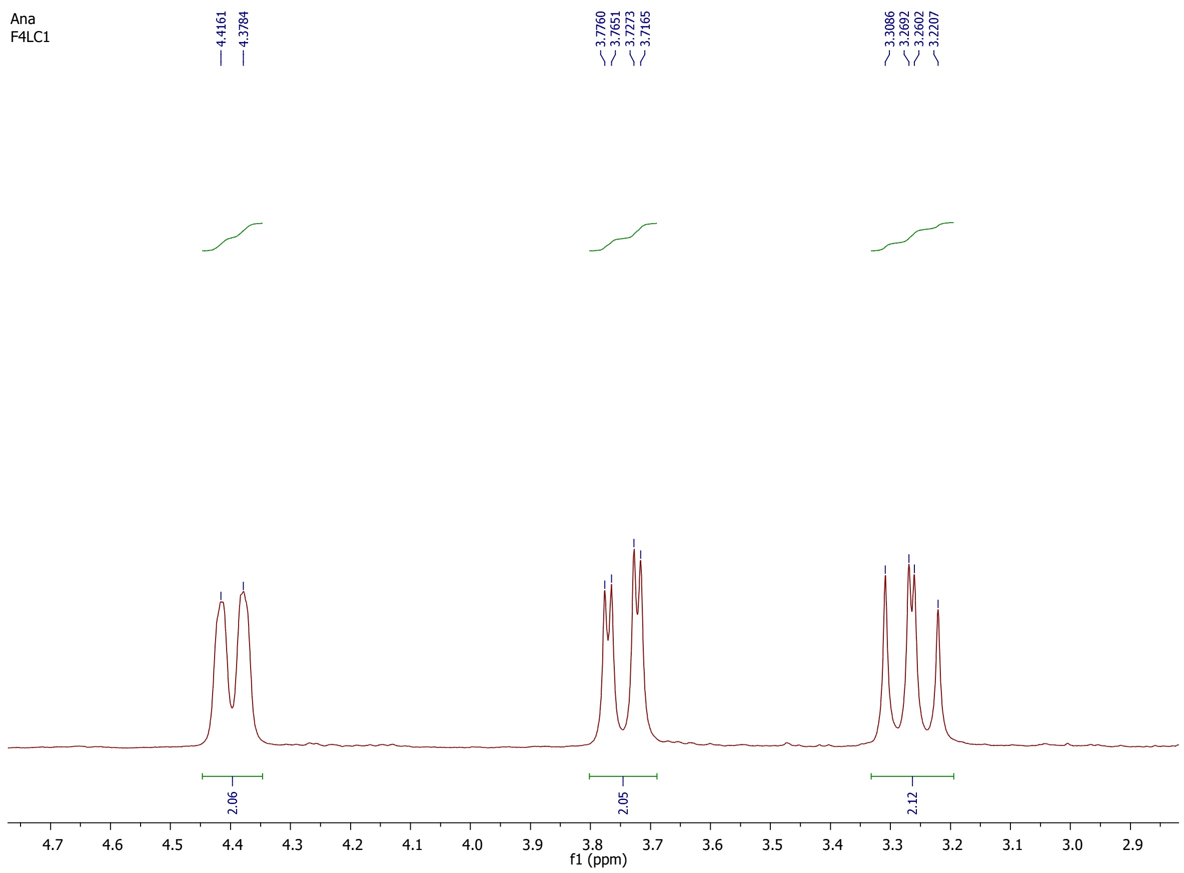

Figure S21: Magnified region from the 1H NMR spectrum of fellutanine C (10) (300 MHz, $CDCl_3$).

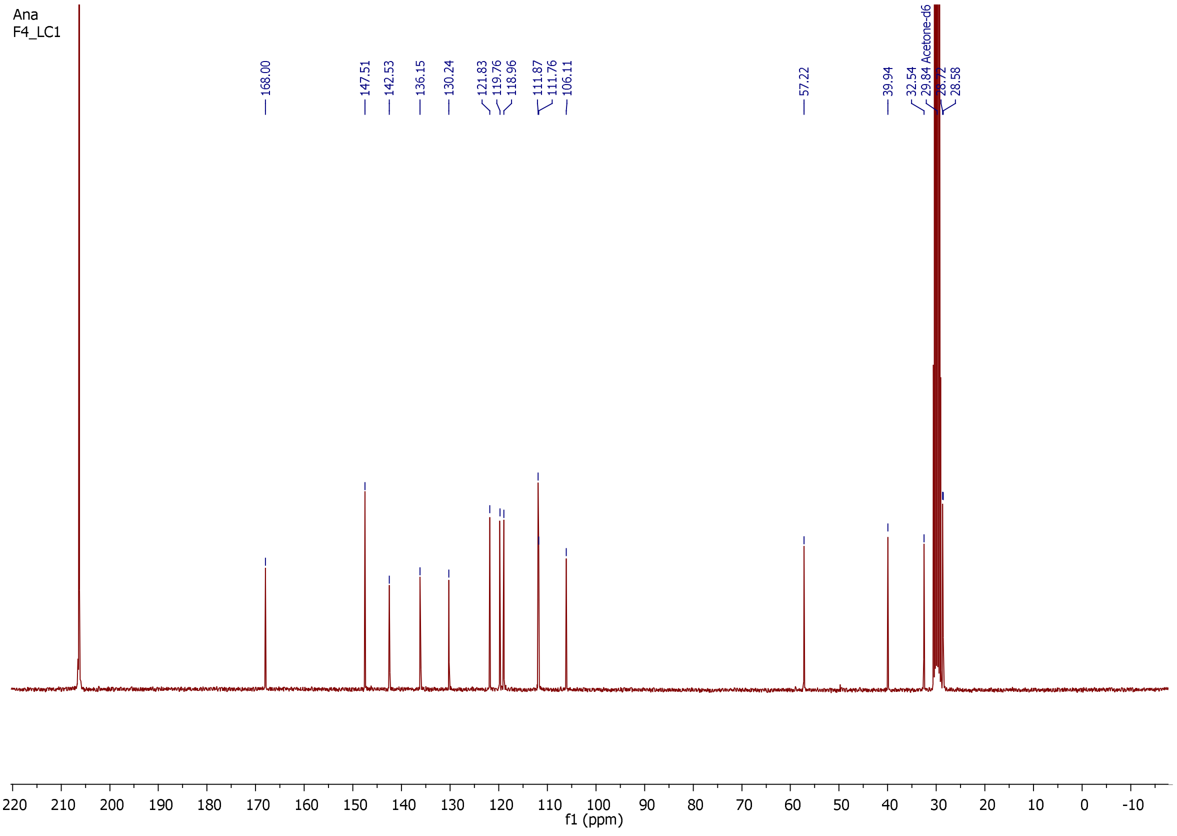

Figure S22: ^{13}C NMR spectrum of fellutanine C (10) (75 MHz, $CDCl_3$).

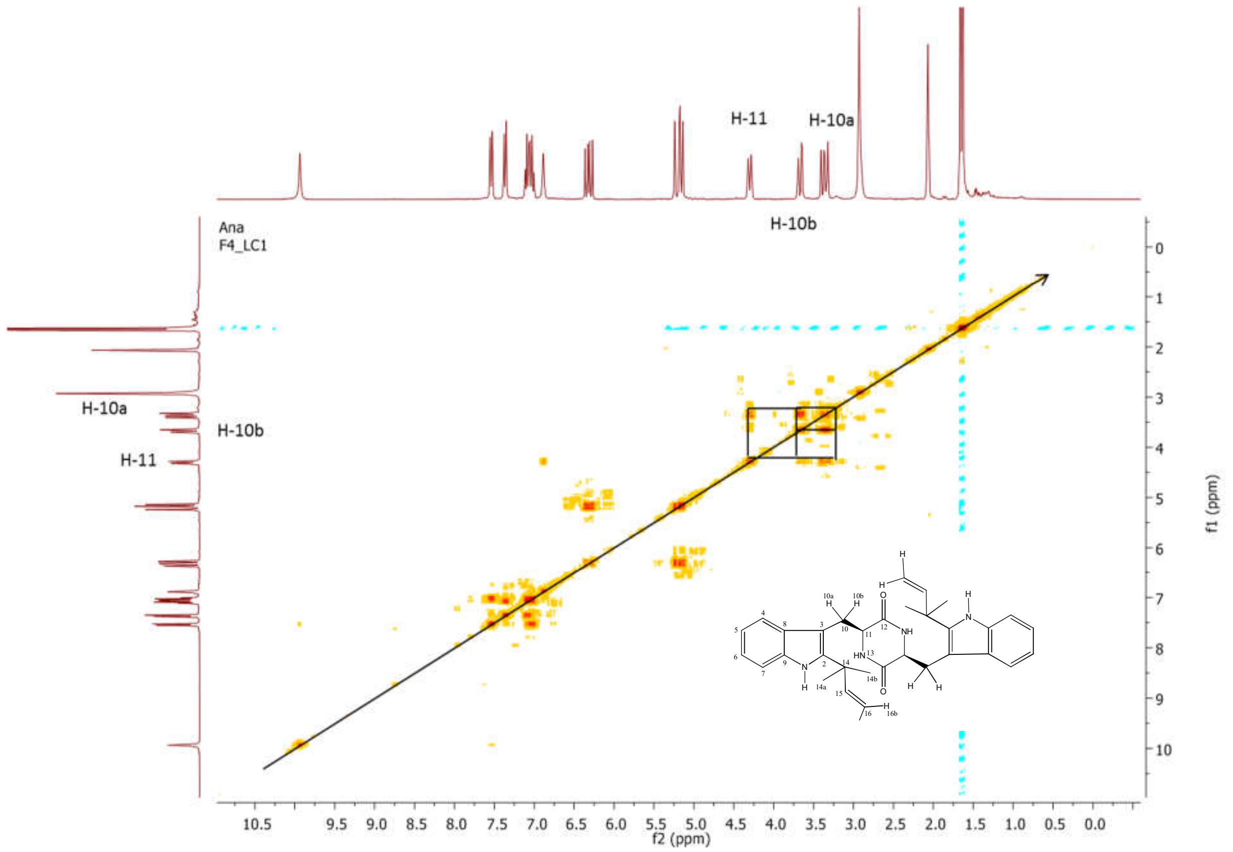

Figure S23: COSY spectrum of fellutanine C (10) (acetone-*d6*)

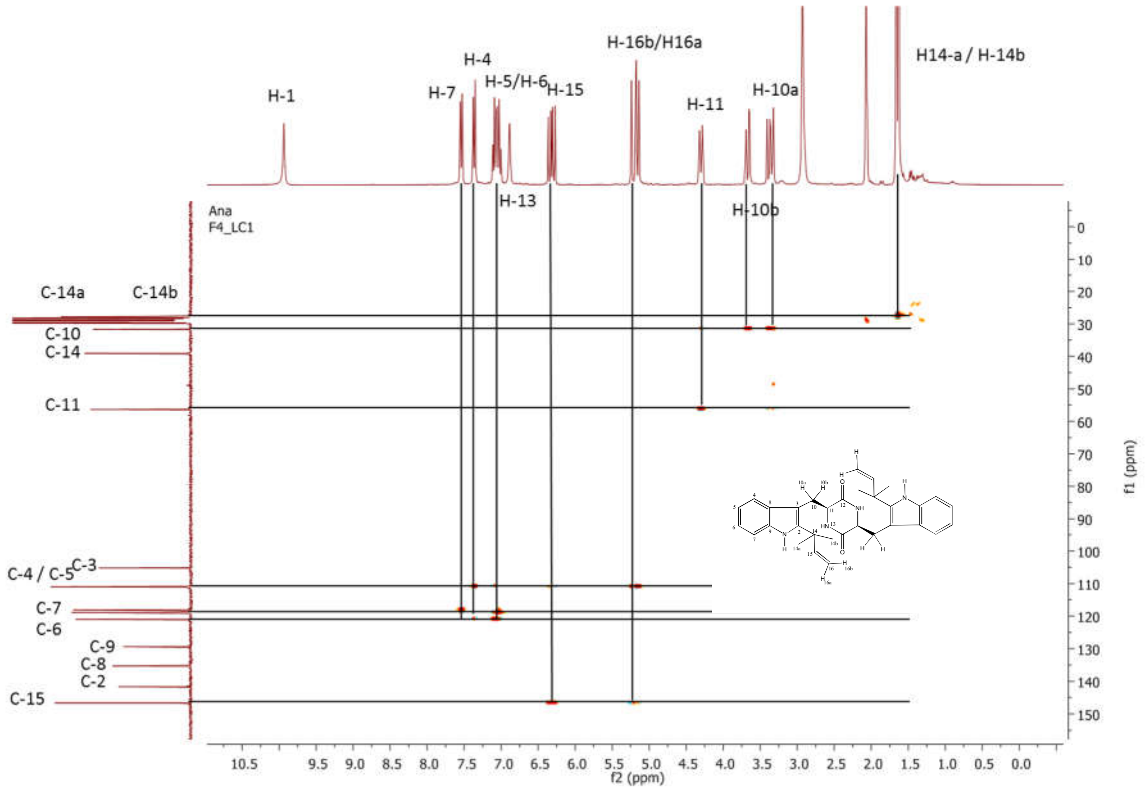

Figure S24: HSQC spectrum of fellutanine C (10) (acetone-*d6*)

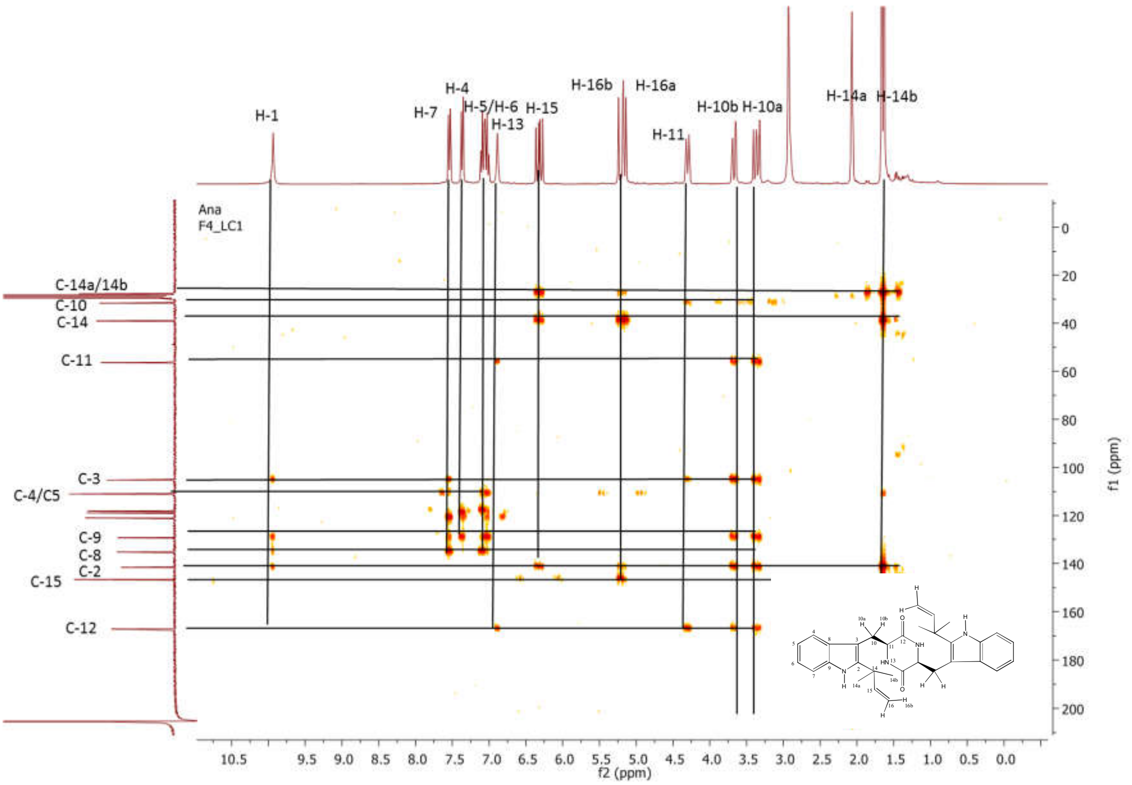

Figure S25: HMBC spectrum of fellutanine C (10) (acetone-*d6*)

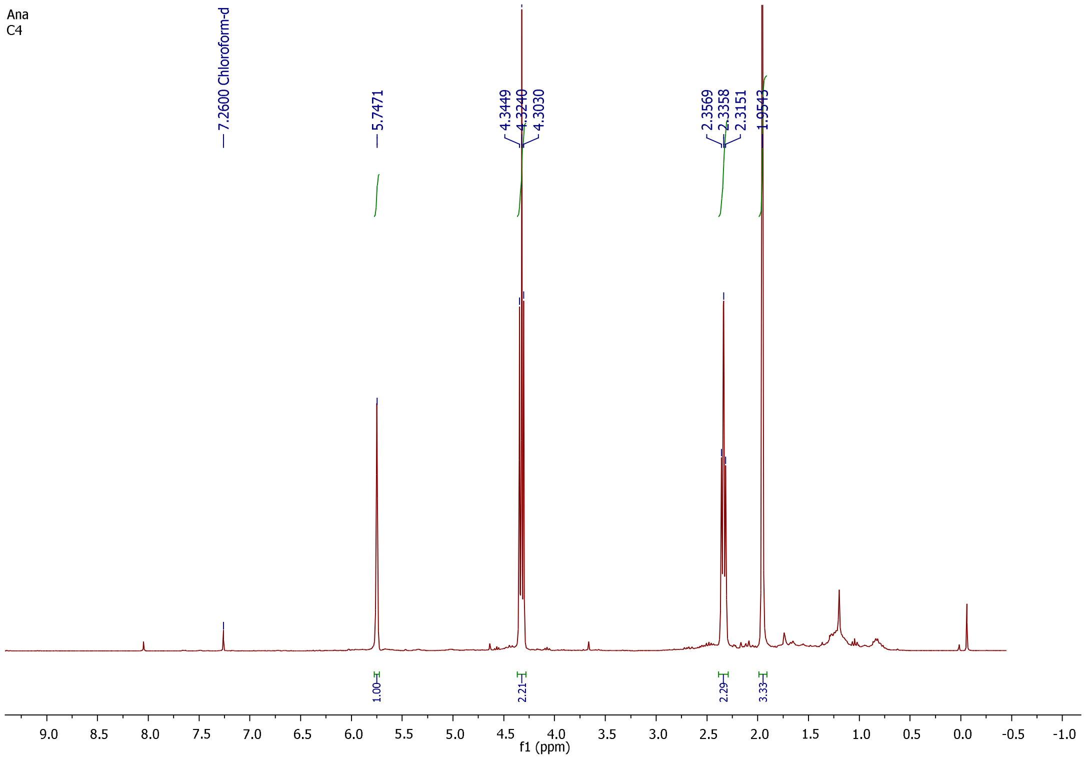

Figure S263: ^{1}H NMR spectrum of 4-methyl-5,6-diihydro-2H-pyran-2-one (15) (300 Hz, $CDCl_3$)

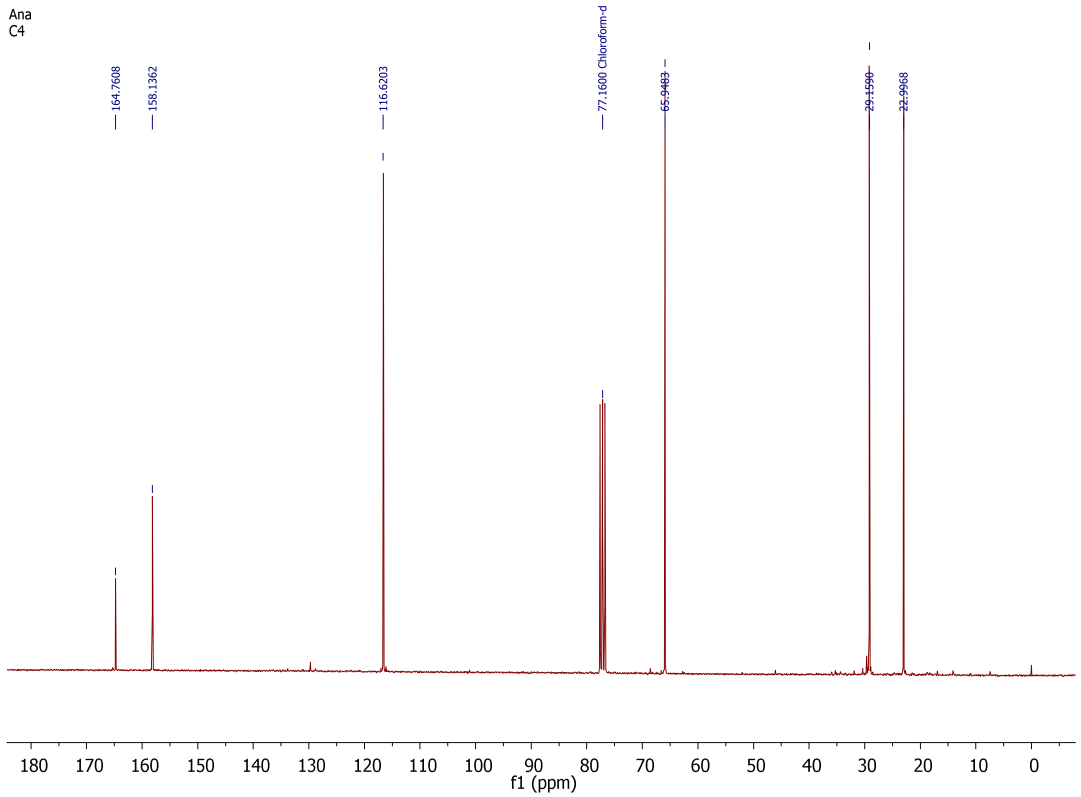

Figure S274: ^{13}C NMR spectrum of 4-methyl-5,6-diihydro-2H-pyran-2-one (15) (75 Hz, $CDCl_3$)

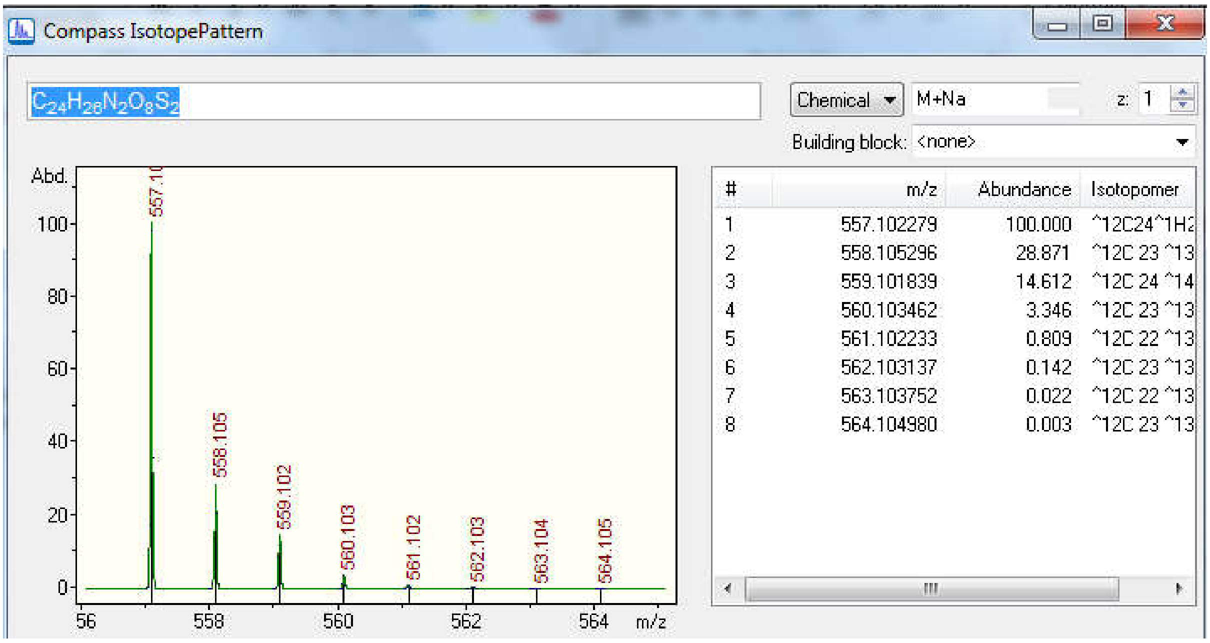

Figure S28: Isotope profile simulation for the compound referring to the accurate mass *m/z* 557.1028 (IsotopePattern – Data Analysis)
